# Multimodal Analgesia With Long-Acting Dinalbuphine Sebacate Plus Transversus Abdominis Plane Block for Perioperative Pain Management in Bariatric Surgery: A Case Report

**DOI:** 10.3389/fphar.2021.683782

**Published:** 2021-05-28

**Authors:** Shih-Yuan Liu, Yi-Hong Ho, Chih-Shung Wong

**Affiliations:** ^1^ School of Medicine, Fu-Jen Catholic University, New Taipei, Taiwan; ^2^ Department of Anesthesiology, Cathay General Hospital, Taipei, Taiwan; ^3^ Graduate Institute of Medical Science, National Defense Medical, Taipei, Taiwan

**Keywords:** bariatric surgery, long-acting dinalbuphine sebacate (naldebain®), TAP, opioid-sparing, MMA

## Abstract

Laparoscopic bariatric surgery is increasingly performed in morbidly obese patients. However, post-surgical pain is common and is usually managed with classical opioids such as morphine and fentanyl. Further, morbidly obese patients are predisposed to opioid-related side effects, especially post-operative nausea and vomiting (PONV), and respiratory depression. Obstructive sleep apnea in morbidly obese patients even predisposes them to respiratory depression. Thus, reducing opioid consumption is important. Multimodal analgesia (MMA) provides optimal perioperative analgesia while minimizing opioid consumption. Studies have shown that MMA strategy can provide sufficient pain relief in bariatric surgery with enhanced recovery. There are very few reports on the use of dinalbuphine sebacate (DS), a newly introduced non-controlled opioid medication with long-lasting analgesic effects. DS has a different mechanism of action from that of morphine or fentanyl and is non-addictive, with minimal side effects. It has been successfully used in laparoscopic cholecystectomy in our previous study. We present a case of a new MMA protocol with DS on a 46-year-old morbidly obese female patient who underwent laparoscopic sleeve gastrectomy. The MMA protocol included ultrasound-guided intramuscular DS injection plus transversus abdominis plane (TAP) block and other analgesics; it achieved good perioperative analgesia with opioid-sparing effect and enhanced patient’s recovery with no pain in the following 4 months.

## Introduction

Bariatric surgery has become a common practice in recent years due to the prevalence of morbid obesity. Currently, the surgery is covered by national health insurance in Taiwan. The laparoscopic approach is frequently preferred due to its minimal invasiveness and less postoperative pain (Weingarten et al., 2011; Alvarez et al., 2014; Ng et al., 2017). However, 41% of patients reported severe pain (7 of 10 in VAS score) in the first 48 h after laparoscopic bariatric surgery (Weingarten et al., 2011). For post-surgical pain, classical systemic opioids such as morphine and fentanyl are still the therapeutic mainstay (Wu et al., 2002; Macrae, 2008; Huang et al., 2018). Obese patients, however, are susceptible to opioid-related side effects, especially post-operative nausea and vomiting (PONV), and respiratory depression (Weingarten et al., 2011; Alvarez et al., 2014; Ng et al., 2017). Further, obstructive sleep apnea is common in morbidly obese patients, which even predisposes them to respiratory depression (Weingarten et al., 2011). Thus, the consumption of classical opioids should be kept at a minimal level. Multimodal analgesia (MMA), which aims to reduce opioid use, has been shown to provide satisfactory perioperative pain relief with an opioid-sparing effect (Alvarez et al., 2014; Ng et al., 2017). With adequate perioperative pain control, the patient’s recovery is enhanced, and potential complications become preventable. For example, with early mobilization, and early discharge, the risk of thrombosis is reduced (Alvarez et al., 2014). Many studies applied multimodal analgesia (MMA) in bariatric surgery and showed good outcomes, but few included dinalbuphine sebacate (DS, Naldebain®) (Alvarez et al., 2014; Ng et al., 2017). Developed based on distinctive non-controlled opioid DS, an extended-release form of nalbuphine, provides long-term (up to 7 days) moderate pain control with no addiction potential and fewer adverse effects. To reduce opioid-related adverse effects and the risk of long-term dependence and abuse, replacing morphine with DS may be an ideal strategy. We particularly implemented DS in our MMA protocol for perioperative pain management in bariatric surgery. On top of that, we adopted the transversus abdominis plane (TAP) block, which is preferred for its low risk of complications, and successful immediate nerve blockade for abdominal surgery pain management. The MMA protocol included ultrasound-guided intramuscular DS injection plus TAP block and other analgesics to achieve good postoperative analgesia with an opioid-sparing effect.

## Case Report

A 46-year-old female patient was diagnosed with morbid obesity (BMI: 43.7 kg/m^2^, Height: 157.5 cm, and Body Weight: 108.4 kg) and treated with insulin and oral hypoglycemic agent for type 2 diabetes mellitus. She was arranged for laparoscopic gastrectomy by sleeve technique (with intra-abdominal pressure of 12 cm H_2_O by CO_2_ insufflation). In preoperative anesthesia assessment, there were no clinically significant findings in routine laboratory examination, electrocardiogram, and chest X-ray. The American Society of Anesthesiologists (ASA) was graded as Class III. No premedication was prescribed and no medical history contradicted with nalbuphine. Vital signs were normal and monitored during the operation. General anesthesia was induced by glycopyrrolate 0.2 mg, 2% lidocaine 50 mg, propofol 200 mg, fentanyl 250 μg. Rocuronium 60 mg was used to facilitate endotracheal intubation with a 7.5 mm inner diameter. Ketamine 50 mg (Remérand et al., 2009; Brinck et al., 2018) and dexamethasone 10 mg (Wang et al., 2011; DREAMS Trial Collaborators and West Midlands Research Collaborative, 2017; Brown et al., 2018) were also given for preemptive analgesia and postoperative nausea and vomiting (PONV) prevention respectively. After the anesthesia induction setup, the patient was placed in a supine position and received one dose of long-acting dinalbuphine sebacate (150 mg DS/2ml, Naldebain® ER Injection, Lumosa Therapeutics Co. Ltd., Taiwan) by ultrasound-guided intramuscular injection at the left upper arm. Anesthesia was maintained by desflurane during the operation. The patient was kept hemodynamically stable throughout the operation.

After the last closing suture was finished, an ultrasound-guided transversus abdominis plane (TAP) block was followed. The patient was placed in a supine position. The convex probe was utilized from the xyphoid process obliquely and laterally along the costal margin. Three muscle layers of transversus abdominis, internal oblique, and external oblique can be distinctly identified by the transducer. The injection site was chosen at the mid-axillary line between the iliac crest and costal margin. It was visualized between the internal oblique and transversus abdominis, and confirmed by hydro-dissection with 5 ml of normal saline, followed by 20 ml 0.5% lidocaine and adjunct fentanyl 100 μg. A total dose of 250 μg fentanyl was administered i.v during the maintenance of the whole procedure and the whole operation lasted less than 2 h. Sugammadex 200 mg was given for residual muscle relaxant reversal and the endotracheal tube was removed smoothly after the patient regained her spontaneous breathing with TOF >0.9, and then the patient was transferred to the postoperative anesthesia care unit (PACU).

Acetaminophen 1 gm and ketorolac 30 mg were given immediately on arrival at the PACU. No pain and PONV were reported during one-hour of PACU stay. Then, the patient was sent to the general ward with a stable condition for further postoperative care. At 3 h after surgery, mild wound pain was noted with 2 out of 10 in the visual analog score. On post-operation day 3, the patient tolerated oral water intake. During the following hospital stay, the patient recovered well, and no further analgesics were given at the ward. The patient was discharged on day 4 after surgery.

From discharge to the first follow-up visit (6 days after surgery), satisfying pain relief (VAS score was below 3) was reported and no analgesics were taken. In the four-month follow-up visit, no analgesic was prescribed and no post-surgical complication was reported. This case report was reviewed and approved by Cathay General Hospital IRB (CGH-IRB No.CGH-P110009) with the patient’s consent.

## Discussions

**FIGURE 1:**
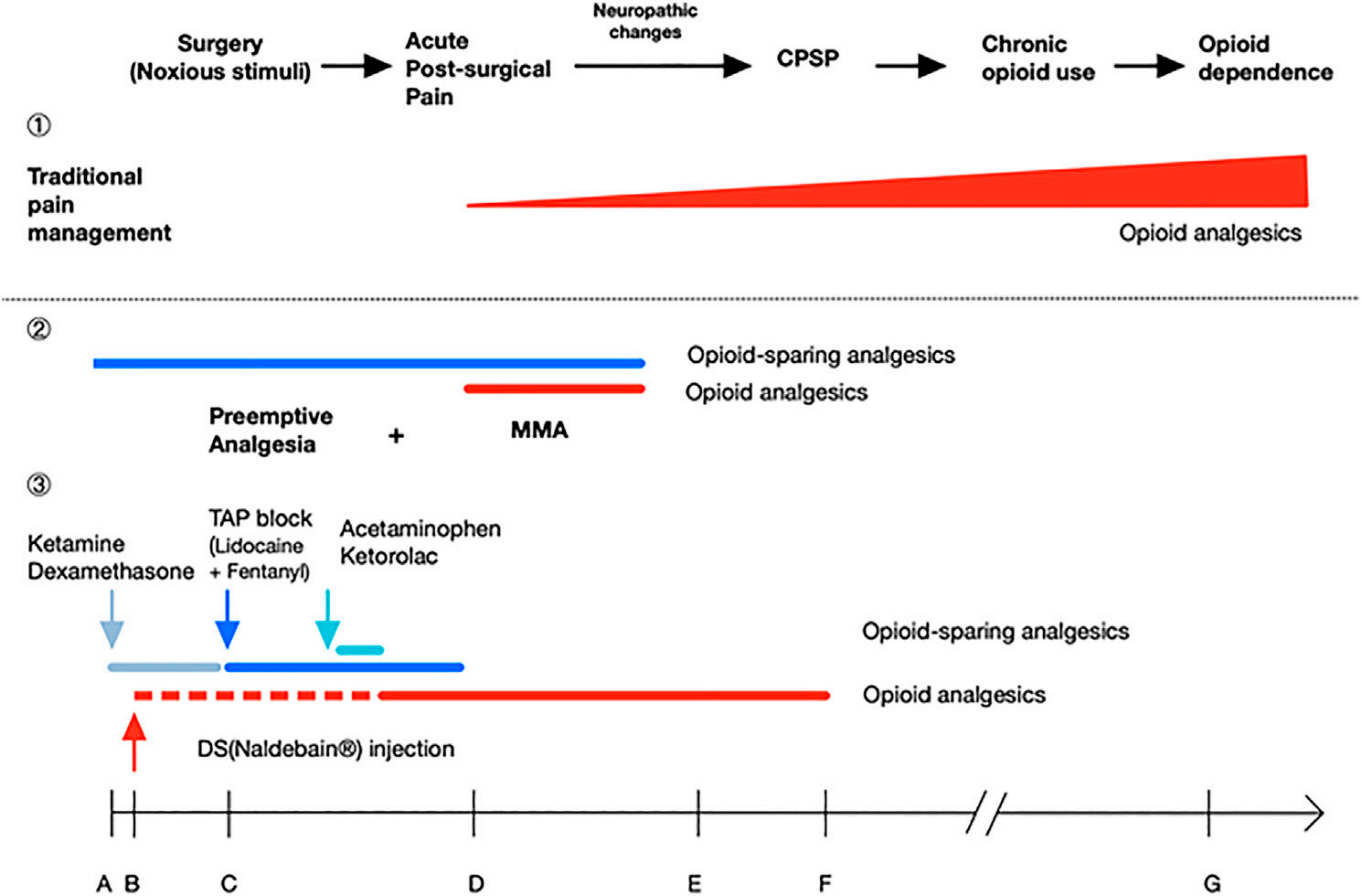
Poorly-managed acute post-surgical pain may lead to neuropathic changes and cause chronic post-surgical pain (CPSP), chronic opioid use and even opioid dependence. ①In traditional pain management, analgesics are given after acute post-surgical pain reported. Further, over-reliance on opioids leads to chronic opioid use and dose escalating. ②Preemptive analgesia aims to prevent post-surgical pain at the very beginning. When it combines with continuous multimodal analgesia (MMA) by adding opioid-sparing agents, the protocol offers balanced analgesia with lesser dose of opioids, thus reducing the risk of CPSP, chronic opioid use, and opioid dependence. ③As an example, this case report shows a successful MMA protocol; it promoted the patient’s recovery without any pain prescriptions, especially narcotics. Ketamine, dexamethasone, TAP block with lidocaine plus fentanyl, acetaminophen, and ketorolac are included to achieve opioid-sparing effect. For DS (naldebain®), an extended-release non-controlled distinguishing opioid, it provided a duration up to 7 days with no addiction potential, and less opioid-related adverse effects. The dotted line stands for 12–24 h for DS to reach the therapeutic nalbuphine level; A ∼ G indicates the time for analgesic administration and duration of action: A, GA induction; B, start of surgery (incision); C, end of surgery; D, 1 day after surgery; E, discharge on day 4 after surgery; F, 7 days after surgery; G, 4 months after surgery.

For the MMA protocol, in this case, we included extended-released DS intramuscular injection before the operation, ultrasound-guided TAP at the end of the surgery, and other pain medications: ketamine, dexamethasone, acetaminophen, and ketorolac for opioid-sparing effect (Alvarez et al., 2014; Remérand et al., 2009; Brinck et al., 2018; Wang et al., 2011; DREAMS Trial Collaborators and West Midlands Research Collaborative, 2017; Tolska et al., 2019). We observed sufficient pain relief without the requirement of other pain medications after discharge. Figure 1. shows the theoretical effect of the MMA on post-surgical recovery, chronic post-surgical pain (CPSP) development, and the later chronic opioid use; as long as successful MMA protocol can be implemented using this case as an example. Ketamine and dexamethasone were given intravenously together with anesthesia induction. Ketamine, an NMDA receptor antagonist, provides an opioid-sparing effect to reduce postoperative opioid consumption, prolongs postoperative analgesia while also slightly reducing the incidence of PONV (Remérand et al., 2009; Alvarez et al., 2014; Ng et al., 2017; Brinck et al., 2018; Tolska et al., 2019). Dexamethasone is recommended as the first-line drug for PONV prevention with no adverse effects and also plays a role in MMA (Wang et al., 2011; Alvarez et al., 2014; DREAMS Trial Collaborators and West Midlands Research Collaborative, 2017), while PONV is the major side effect of volatile general anesthesia and opioid use.

Ultrasound-guided intramuscular long-acting DS was given immediately after general anesthesia induction. DS is an extended-release prodrug of nalbuphine which acts as a partial agonist at the *κ*-opioid receptor and a weak antagonist at the µ-opioid receptor; it is slightly less potent than morphine. Compared to morphine, DS produces less dependence, less sedation, and less respiratory depression (Yeh et al., 2017; Lee et al., 2020). DS (Naldebain® Injection, Lumosa Therapeutics, Taiwan) was introduced in Taiwan in 2017; it, *via* intramuscular injection, delivers and maintains an effective nalbuphine analgesic blood level for approximately 7 days (Huang et al., 2020). Our previous study showed DS provided satisfying postoperative pain relief for laparoscopic cholecystectomy (Lee et al., 2020). The postoperative pain scores of laparoscopic bariatric surgery are variable from 6 to 8 out of 10 (Gerbershagen et al., 2013; Brummett et al., 2017).

The recommended injecting time of DS is 12–24 h before surgery to attain peak plasma concentration, providing preemptive analgesia, and continuous postoperative pain control for another 5–6 days. However, injection site pain is the most reported complication, and the incidence rate was up to 61% (Wang et al., 2011). In this case, the patient was admitted just before surgery on the day of the operation. As expected, the time for DS to take effect was not sufficient as it needs 12–24 h to reach peak analgesic effect concentration. To resolve this problem, we conducted an ultrasound-guided TAP block for immediate pain control before DS reached the therapeutic blood level; this lidocaine regimen maybe not enough for the maintenance of analgesia over 12 h, however, the presence of 100 μg of fentanyl adjunct may enhance, and prolong the duration of TAP blocking effect. In this case, no gap was observed with satisfactory analgesia after emergence from general anesthesia. On top of that, the adipose layer of this patient was so thick (more than 5 cm). It was hard to inject DS into the gluteal muscle following the manufacture’s insert recommended; accidental DS injection into the adipose layer might cause the oil-based DS formulation to fail to release into the blood vessel and even cause a painful lipid pack in the subcutaneous or adipose tissue as reported injection pain. To avoid the aforementioned incident and correctly inject DS into muscle, we injected DS into the left upper arm by ultrasound guidance.

At the end of the surgery before anesthesia emergence, TAP block was conducted with lidocaine (0.5% 20 ml) plus fentanyl (100 μg), to provide immediate postoperative analgesia before the DS can reach effective blood concentration (Kearns and Young, 2011; Sammons and Ritchey, 2015). TAP block is an abdominal wall field block targeting the ventral rami of the T7 ∼ L1 spinal nerves. By acting peripherally, it completely prevents noxious stimuli from reaching the central nervous system. Several studies demonstrated that TAP blocks yielded adequate analgesia and could reduce opioid consumption in the first 24 h after abdominal surgery (Kearns and Young, 2011; Sammons and Ritchey, 2015). For the future application of MMA with TAP block, however, a larger volume of longer-lasting local anesthetic (levobupivacaine or ropivacaine) would certainly provide a better spread effect, and ensure more effective pain control.

Ketorolac 30 mg and acetaminophen 1 gm were given on arrival of PACU. As known, non-selective cyclooxygenase inhibitor ketorolac has been demonstrated to provide good efficacy in postoperative analgesia. Its mean plasma half-life is approximately 5.5 h and can reach maximum effect by 1 or 2 h by oral, for intravenous injection, it produces an immediate pain relief at PAUC with a duration of 4–6 h (Gillis and Brogden, 1997). In a study by Cassinelli et al., 2008, ketorolac has been shown to decrease visual analog pain score if administered immediately postoperatively. It is recommended as a component of multimodal analgesia in obese patients with opioid-sparing effects (Alvarez et al., 2014). As for acetaminophen, N-acetyl-para-aminophenol, it can centrally inhibit prostaglandin synthesis by inhibiting both COX-1 and COX-2 enzymes activity with no sedative effect, and is also shown to possess a significant opioid-sparing effect (White et al., 2007); it has been widely utilized in postoperative analgesia, as a part of MMA in ERAS protocol in our hospital, due to its rapid onset for immediate pain control (Brown et al., 2018). Both ketorolac and acetaminophen are standard rescue analgesics at our PACU, which provide opioid-sparing effect and satisfactory pain relief.

## Conclusions

Classical opioid use such as morphine or fentanyl should be reduced in surgery, especially in obese patients for avoiding PONV and respiratory depression. MMA strategy is widely used in anesthesia practice and perioperative analgesia to minimize opioid consumption with opioid-sparing effect, particularly in bariatric surgery. DS (naldebain®) is a newly-introduced non-controlled opioid medication with a long duration and no addiction potential. We demonstrated a successful MMA protocol with DS on this obese patient undergoing laparoscopic sleeve gastrectomy; it included ultrasound-guided intramuscular DS (150 mg, not a body weight-related dose) injection plus TAP block and other analgesics (ketorolac and acetaminophen). From this case, we suggest the slow release of nalbuphine, DS, may play an important role in perioperative MMA. A large-scale clinical trial is needed to show its role in the MMA regimen.

## Data Availability

The datasets presented in this article are not readily available because No dataset. Requests to access the datasets should be directed to C-SW, w82556@gmail.com

## References

[B1] Alvarez A. Singh P. M. Sinha A. C. (2014). Postoperative Analgesia in Morbid Obesity. Obes. Surg. 24, 652–659. 10.1007/s11695-014-1185-2 24431032

[B2] Brinck E. C. Tiippana E. Heesen M. Bell R. F. Straube S. Moore R. A. (2018). Perioperative Intravenous Ketamine for Acute Postoperative Pain in Adults. Cochrane Database Syst. Rev. 12, Cd012033. 30570761 10.1002/14651858.CD012033.pub4 PMC6360925

[B3] Brown E. N. Pavone K. J. Naranjo M. (2018). Multimodal General Anesthesia. Anesth. Analgesia 127, 1246–1258. 10.1213/ane.0000000000003668 PMC6203428 30252709

[B5] Brummett C. M. Waljee J. F. Goesling J. Moser S. Lin P. Englesbe M. J. (2017). New Persistent Opioid Use After Minor and Major Surgical Procedures in US Adults. JAMA Surg. 152, e170504. 10.1001/jamasurg.2017.0504 28403427 PMC7050825

[B6] Cassinelli E. H. Dean C. L. Garcia R. M. Furey C. G. Bohlman H. H. (2008). Ketorolac Use for Postoperative Pain Management Following Lumbar Decompression Surgery: A Prospective, Randomized, Double-Blinded, Placebo-Controlled Trial. Spine (Phila Pa 1976) 33, 1313–1317. 10.1097/BRS.0b013e31817329bd 18496342

[B7] DREAMS Trial Collaborators and West Midlands Research Collaborative (2017). Dexamethasone versus Standard Treatment for Postoperative Nausea and Vomiting in Gastrointestinal Surgery: Randomised Controlled Trial (DREAMS Trial). Bmj 357, j1455. 10.1136/bmj.j1455 28420629 PMC5482348

[B8] Gerbershagen H. J. Aduckathil S. van Wijck A. J. M. Peelen L. M. Kalkman C. J. Meissner W. (2013). Pain Intensity on the First Day After Surgery. Anesthesiology 118, 934–944. 10.1097/aln.0b013e31828866b3 23392233

[B9] Gillis J. C. Brogden R. N. (1997). Ketorolac. Drugs 53, 139–188. 10.2165/00003495-199753010-00012 9010653

[B10] Huang C. C. Sun W. Z. Wong C. S. (2018). Prevention of Chronic Postsurgical Pain: The Effect of Preventive and Multimodal Analgesia. Asian J. Anesthesiol 56, 74–82. 10.6859/aja.201809_56(3).0002 30583329

[B11] Huang W.-H. Huang N.-C. Lin J.-A. Wong C.-S. (2020). Multimodal Analgesia for Shoulder Rotator Cuff Surgery Pain: The Role of Naldebain® and Ultrasound-Guided Peripheral Nerve Blocks Combination. J. Med. Sci. 40, 279–283.

[B12] Kearns R. J. Young S. J. (2011). Transversus Abdominis Plane Blocks; a National Survey of Techniques Used by UK Obstetric Anaesthetists. Int. J. Obstet. Anesth. 20, 103–104. 10.1016/j.ijoa.2010.08.005 21112766

[B13] Lee S.-O. Huang L.-P. Wong C.-S. (2020). Preoperative Administration of Extended-Release Dinalbuphine Sebacate Compares with Morphine for Post-Laparoscopic Cholecystectomy Pain Management: A Randomized Study. Jpr 13, 2247–2253. 10.2147/jpr.s263315 PMC7490073 32982387

[B14] Macrae W. A. (2008). Chronic post-surgical Pain: 10 Years on. Br. J. Anaesth. 101, 77–86. 10.1093/bja/aen099 18434337

[B15] Ng J. J. Leong W. Q. Tan C. S. Poon K. H. Lomanto D. So J. B. Y. (2017). A Multimodal Analgesic Protocol Reduces Opioid-Related Adverse Events and Improves Patient Outcomes in Laparoscopic Sleeve Gastrectomy. Obes. Surg. 27, 3075–3081. 10.1007/s11695-017-2790-7 28674840

[B16] Remérand F. Le Tendre C. Baud A. Couvret C. Pourrat X. Favard L. (2009). The Early and Delayed Analgesic Effects of Ketamine after Total Hip Arthroplasty: A Prospective, Randomized, Controlled, Double-Blind Study. Anesth. Analgesia 109, 1963–1971. 10.1213/ane.0b013e3181bdc8a0 19923527

[B17] Sammons G. Ritchey W. (2015). Use of Transversus Abdominis Plane (TAP) Blocks for Pain Management in Elderly Surgical Patients. AORN J. 102, 493–497. 10.1016/j.aorn.2015.09.003 26514706

[B18] Tolska H. K. Hamunen K. Takala A. Kontinen V. K. (2019). Systematic Review of Analgesics and Dexamethasone for Post-Tonsillectomy Pain in Adults. Br. J. Anaesth. 123, e397–e411. 10.1016/j.bja.2019.04.063 31221427 PMC6676167

[B19] Wang F.-Y. Shen Y.-C. Chen M.-K. Chau S.-W. Ku C.-L. Feng Y.-T. (2011). Equal Volumes of Undiluted Nalbuphine and Lidocaine and normal Diluted Saline Prevents Nalbuphine-Induced Injection Pain. Acta Anaesthesiologica Taiwanica 49, 125–129. 10.1016/j.aat.2011.11.009 22221683

[B20] Weingarten T. N. Sprung J. Flores A. Oviedo Baena A. M. Schroeder D. R. Warner D. O. (2011). Opioid Requirements after Laparoscopic Bariatric Surgery. Obes. Surg. 21, 1407–1412. 10.1007/s11695-010-0217-9 20563662

[B21] White P. F. Kehlet H. Neal J. M. Schricker T. Carr D. B. Carli F. S.S. Group (2007). The Role of the Anesthesiologist in Fast-Track Surgery: From Multimodal Analgesia to Perioperative Medical Care. Anesth. Analgesia 104, 1380–1396. 10.1213/01.ane.0000263034.96885.e1 17513630

[B22] Wu C. L. Berenholtz S. M. Pronovost P. J. Fleisher L. A. (2002). Systematic Review and Analysis of Postdischarge Symptoms after Outpatient Surgery. Anesthesiology 96, 994–1003. 10.1097/00000542-200204000-00030 11964610

[B23] Yeh C.-Y. Jao S.-W. Chen J.-S. Fan C.-W. Chen H.-H. Hsieh P.-S. (2017). Sebacoyl Dinalbuphine Ester Extended-Release Injection for Long-Acting Analgesia. Clin. J. Pain 33, 429–434. 10.1097/ajp.0000000000000417 27518486

